# Making sense of Cronbach's alpha

**DOI:** 10.5116/ijme.4dfb.8dfd

**Published:** 2011-06-27

**Authors:** Mohsen Tavakol, Reg Dennick

**Affiliations:** The University of Nottingham, Medical Education Unit, UK; Correspondence: Mohsen Tavakol, The University of Nottingham, Medical Education Unit, UK; E-mail: mohsen.tavakol@ijme.net

Medical educators attempt to create reliable and valid tests and questionnaires in order to enhance the accuracy of their assessment and evaluations. Validity and reliability are two fundamental elements in the evaluation of a measurement instrument. Instruments can be conventional knowledge, skill or attitude tests, clinical simulations or survey questionnaires. Instruments can measure concepts, psychomotor skills or affective values. Validity is concerned with the extent to which an instrument measures what it is intended to measure. Reliability is concerned with the ability of an instrument to measure consistently.^1^ It should be noted that the reliability of an instrument is closely associated with its validity. An instrument cannot be valid unless it is reliable. However, the reliability of an instrument does not depend on its validity.^2^ It is possible to objectively measure the reliability of an instrument and in this paper we explain the meaning of Cronbach’s alpha, the most widely used objective measure of reliability.

Calculating alpha has become common practice in medical education research when multiple-item measures of a concept or construct are employed. This is because it is easier to use in comparison to other estimates (e.g. test-retest reliability estimates)^3^ as it only requires one test administration. However, in spite of the widespread use of alpha in the literature the meaning, proper use and interpretation of alpha is not clearly understood. ^2^^, ^^4^^, ^^5^ We feel it is important, therefore, to further explain the underlying assumptions behind alpha in order to promote its more effective use. It should be emphasised that the purpose of this brief overview is just to focus on Cronbach’s alpha as an index of reliability. Alternative methods of measuring reliability based on other psychometric methods, such as generalisability theory or item-response theory, can be used for monitoring and improving the quality of OSCE examinations ^6^^-^^10^, but will not be discussed here.

## What is Cronbach alpha?

Alpha was developed by Lee Cronbach in 1951^11^ to provide a measure of the internal consistency of a test or scale; it is expressed as a number between 0 and 1. Internal consistency describes the extent to which all the items in a test measure the same concept or construct and hence it is connected to the inter-relatedness of the items within the test. Internal consistency should be determined before a test can be employed for research or examination purposes to ensure validity. In addition, reliability estimates show the amount of measurement error in a test. Put simply, this interpretation of reliability is the correlation of test with itself. Squaring this correlation and subtracting from 1.00 produces the index of measurement error. For example, if a test has a reliability of 0.80, there is 0.36 error variance (random error) in the scores (0.80×0.80 = 0.64; 1.00 – 0.64 = 0.36).^12^ As the estimate of reliability increases, the fraction of a test score that is attributable to error will decrease.^2^ It is of note that the reliability of a test reveals the effect of measurement error on the observed score of a student cohort rather than on an individual student. To calculate the effect of measurement error on the observed score of an individual student, the standard error of measurement must be calculated (SEM).^13^

If the items in a test are correlated to each other, the value of alpha is increased. However, a high coefficient alpha does not always mean a high degree of internal consistency. This is because alpha is also affected by the length of the test. If the test length is too short, the value of alpha is reduced.^2^^, ^^14^ Thus, to increase alpha, more related items testing the same concept should be added to the test. It is also important to note that alpha is a property of the scores on a test from a specific sample of testees. Therefore investigators should not rely on published alpha estimates and should measure alpha each time the test is administered.^14^

## Use of Cronbach’s alpha

Improper use of alpha can lead to situations in which either a test or scale is wrongly discarded or the test is criticised for not generating trustworthy results. To avoid this situation an understanding of the associated concepts of internal consistency, homogeneity or unidimensionality can help to improve the use of alpha. Internal consistency is concerned with the interrelatedness of a sample of test items, whereas homogeneity refers to unidimensionality. A measure is said to be unidimensional if its items measure a single latent trait or construct. Internal consistency is a necessary but not sufficient condition for measuring homogeneity or unidimensionality in a sample of test items. ^5^^, ^^15^ Fundamentally, the concept of reliability assumes that unidimensionality exists in a sample of test items^16^ and if this assumption is violated it does cause a major underestimate of reliability. It has been well documented that a multidimensional test does not necessary have a lower alpha than a unidimensional test. Thus a more rigorous view of alpha is that it cannot simply be interpreted as an index for the internal consistency of a test. ^5^^, ^^15^^, ^^17^

Factor Analysis can be used to identify the dimensions of a test.^18^ Other reliable techniques have been used and we encourage the reader to consult the paper “Applied Dimensionality and Test Structure Assessment with the START-M Mathematics Test” and to compare methods for assessing the dimensionality and underlying structure of a test.^19^

Alpha, therefore, does not simply measure the unidimensionality of a set of items, but can be used to confirm whether or not a sample of items is actually unidimensional.^5^ On the other hand if a test has more than one concept or construct, it may not make sense to report alpha for the test as a whole as the larger number of questions will inevitable inflate the value of alpha. In principle therefore, alpha should be calculated for each of the concepts rather than for the entire test or scale. ^2^^, ^^3^ The implication for a summative examination containing heterogeneous, case-based questions is that alpha should be calculated for each case.

More importantly, alpha is grounded in the ‘tau equivalent model’ which assumes that each test item measures the same latent trait on the same scale. Therefore, if multiple factors/traits underlie the items on a scale, as revealed by Factor Analysis, this assumption is violated and alpha underestimates the reliability of the test.^17^ If the number of test items is too small it will also violate the assumption of tau-equivalence and will underestimate reliability.^20^ When test items meet the assumptions of the tau-equivalent model, alpha approaches a better estimate of reliability. In practice, Cronbach’s alpha is a lower-bound estimate of reliability because heterogeneous test items would violate the assumptions of the tau-equivalent model.^5^ If the calculation of “standardised item alpha” in SPSS is higher than “Cronbach’s alpha”, a further examination of the tau-equivalent measurement in the data may be essential.

## Numerical values of alpha

As pointed out earlier, the number of test items, item inter-relatedness and dimensionality affect the value of alpha.^5^ There are different reports about the acceptable values of alpha, ranging from 0.70 to 0.95. ^2^^, ^^21^^, ^^22^ A low value of alpha could be due to a low number of questions, poor inter-relatedness between items or heterogeneous constructs. For example if a low alpha is due to poor correlation between items then some should be revised or discarded. The easiest method to find them is to compute the correlation of each test item with the total score test; items with low correlations (approaching zero) are deleted. If alpha is too high it may suggest that some items are redundant as they are testing the same question but in a different guise. A maximum alpha value of 0.90 has been recommended.^14^

## Summary

High quality tests are important to evaluate the reliability of data supplied in an examination or a research study. Alpha is a commonly employed index of test reliability. Alpha is affected by the test length and dimensionality. Alpha as an index of reliability should follow the assumptions of the essentially tau-equivalent approach. A low alpha appears if these assumptions are not meet. Alpha does not simply measure test homogeneity or unidimensionality as test reliability is a function of test length. A longer test increases the reliability of a test regardless of whether the test is homogenous or not. A high value of alpha (> 0.90) may suggest redundancies and show that the test length should be shortened.

## Conclusions

Alpha is an important concept in the evaluation of assessments and questionnaires. It is mandatory that assessors and researchers should estimate this quantity to add validity and accuracy to the interpretation of their data. Nevertheless alpha has frequently been reported in an uncritical way and without adequate understanding and interpretation. In this editorial we have attempted to explain the assumptions underlying the calculation of alpha, the factors influencing its magnitude and the ways in which its value can be interpreted. We hope that investigators in future will be more critical when reporting values of alpha in their studies.
